# Partition-Based Point Cloud Completion Network with Density Refinement

**DOI:** 10.3390/e25071018

**Published:** 2023-07-02

**Authors:** Jianxin Li, Guannan Si, Xinyu Liang, Zhaoliang An, Pengxin Tian, Fengyu Zhou

**Affiliations:** 1 School of Electrical Engineering, Academy of Information Sciences, Shandong Jiaotong University, Jinan 250357, China; 2 School of Control Science and Engineering, Shandong University, Jinan 250012, China

**Keywords:** convolutional neural networks, point cloud completion, gridding, radar, geometric density

## Abstract

In this paper, we propose a novel method for point cloud complementation called PADPNet. Our approach uses a combination of global and local information to infer missing elements in the point cloud. We achieve this by dividing the input point cloud into uniform local regions, called perceptual fields, which are abstractly understood as special convolution kernels. The set of point clouds in each local region is represented as a feature vector and transformed into N uniform perceptual fields as the input to our transformer model. We also designed a geometric density-aware block to better exploit the inductive bias of the point cloud’s 3D geometric structure. Our method preserves sharp edges and detailed structures that are often lost in voxel-based or point-based approaches. Experimental results demonstrate that our approach outperforms other methods in reducing the ambiguity of output results. Our proposed method has important applications in 3D computer vision and can efficiently recover complete 3D object shapes from missing point clouds.

## 1. Introduction

Recently, graph-based convolutional neural networks (GCNNs) [1] approaches have been successful in learning point cloud tasks. State-of-the-art methods such as Point-Net [2], PointNet++ [3], and dynamic graph convolutional neural networks (DGCNNs) [4] aim to recover the topology of point clouds through rich learned representations.

Early point cloud completion schemes attempted to complete the task using 2D features that migrated to 3D feature space with an encoder-decoder structure. However, due to the need to process both 2D image and 3D point cloud data simultaneously, these models may have higher time complexity than models using only one type of data. With the success of the PointNet and PointNet++ models, more researchers are willing to process point cloud data directly. These methods mostly use convolutional operations to process local features in point clouds but may be too conservative in terms of point cloud density. As a result, some fine-grained information may be lost and difficult to recover during the decoding stage due to density differences.

Recent studies have introduced attention mechanisms into Graph Convolutional Networks (GCNs) [5] to recover fine-grained shapes. Wu [6] sampled local regions of inputs and encoded their features to combine with global features. This approach reconstructs high-density complete point clouds from partial point clouds through parallel multiscale feature extraction, cross-region feature fusion, and two-stage feature decoding. The pioneering Point Completion Network (PCN) [7] uses a PointNet-based encoder to generate global features for shape complementation of point clouds. However, it is unable to recover fine geometric details, Subsequent work provided better complementation results by using local features to preserve the geometric details observed in incomplete point shapes. To recover point cloud details and preserve the original planar structure, GCNN-based [8,9,10,11,12,13] methods use deep autoencoders with millions of learnable parameters, making them prone to overfitting. Large-scale labeled datasets are required for training GCNNs for generalizable solutions to shape complementation and classification problems. However, creating labeled ground truth data for point clouds is challenging and expensive. In this paper, we propose a novel approach to address the problems of point cloud data complementation using point cloud partition slicing and density. Our model, named PDC-Net(Partition-Density Completion Network), has three key contributions:

(1) Proposed an encoder-decoder architecture that models pairwise interactions between point cloud elements to infer missing elements for point cloud completion.
(2) Introduced a spatially sliced sensory field that transforms the input point cloud into N uniform perceptual fields for better local feature representation in the transformer model.
(3) Developed a geometric density-aware block to improve the exploitation of the 3D geometric structure and preserve fine details in the point cloud.

## 2. Related Work

### 2.1. Point Cloud Convolution

Convolutional neural networks [14,15] have achieved impressive results in 2D image applications. However, processing unstructured 3D point cloud data with standard convolutional networks is not feasible. Existing work converts point clouds into regular grid representations, such as voxel grids or multi-view images, for further processing. However, these methods suffer from high memory consumption and computational burden. Sparse representation-based methods alleviate these problems to some extent, but the quantization operations still result in a significant loss of detailed information. Geometry-based methods are effective for feature extraction from point cloud data due to their association with graph neural networks. However, the irregularity of the data makes it difficult to handle. Various GCN [16,17,18] variants with different feature aggregation algorithms have been proposed to improve its performance. These related works demonstrate the challenges of processing unstructured 3D point cloud data [19,20] and the different approaches that have been proposed to tackle this problem.

In contrast to the above approaches, work such as PointNet and PointNet++ involves 3D point clouds and provides an enlightening study of point cloud classification and segmentation tasks. Researchers have transformed point clouds into regular representations, such as voxels or multi-view images, or processed irregular and disordered data by desymbolizing novel convolution kernels and operations.

### 2.2. Point Cloud Completion

Various methods have been proposed for point cloud completion, including view-based, point-based, graph-based, folding-based, and others. Point-based methods model each point independently using multilayer perceptrons (MLPs) [21,22,23] and aggregate global features through symmetric functions [24,25]. Examples include PointNet, PointNet++, and TopNet [26], which use hierarchical structures to consider geometric information. PointNet++ proposes two ensemble abstraction layers to intelligently aggregate multi-layer information, while TopNet proposes a new decoder that can generate structured point clouds without assuming any specific structure or topology. However, the point-based approach has limitations, including a lack of consideration for the geometric information and intercorrelation of the point cloud as a whole, leading to the loss of local features. Additionally, the global-based point embedding method results in the loss of high-frequency information in 3D shapes. Most methods follow a coarse-to-fine approach to improve object localization and details. However, point-on-point sampling methods such as bilinear interpolation, transposed convolution, upsampling, and non-pooling [27,28,29], which are typically used at the end of the model, are difficult to integrate for complex topologies.

Several methods have attempted to use 3D convolutional networks to learn the volumetric representation of 3D point clouds. However, these methods can result in the loss of quantization over feature details and poor representation of fine-grained information due to the characteristics of convolutional networks. 

To overcome these limitations, kPConv [30] was developed. kPConv uses a new point convolution design that operates on a point cloud without any intermediate representation. The convolution weights are localized in Euclidean space by kernel points and applied to input points near the kernel points. kPConv is more flexible than fixed grid convolution since it can use any number of kernel points, while its deformed convolution operator can efficiently learn local displacements and adapt the convolution kernel to the point cloud geometry.

## 3. Method

Our proposed architecture for point cloud completion tasks combines density and partition-based methods to achieve robust results while eliminating unnecessary point cloud overlap and overflow. In the encoder part of our architecture, we utilize both global and local features, extracted from the original point cloud X and a locally divided point cloud, respectively. This allows us to capture both the overall shape and fine-grained details of the object. The global features are obtained using a PointNet-like architecture, which takes the original point cloud as input and outputs a global feature vector. The local features, on the other hand, are obtained by dividing the point cloud into non-overlapping partitions and extracting features from each partition using a local PointNet-like network. These local features are then concatenated with the global features to form the fused feature vector. In the decoder part of our architecture, we use a fully connected layer to generate a fine output point cloud. To further refine the output, we utilize density-based clustering algorithms to remove any remaining noise and improve the structure of the point cloud. We also use inverse density values to weigh the contribution of each point in the output, allowing us to avoid overfitting to high-density regions and underfitting to low-density regions.

To ensure that our architecture is invariant to rotation and translation, we include a transformed network inspired by the T-net structure in PointNet. This network generates an affine transformation matrix to normalize any changes in the point cloud’s orientation or position. The transformed network takes the original point cloud as input and outputs a 3 × 3 rotation matrix.

**Figure 1:**
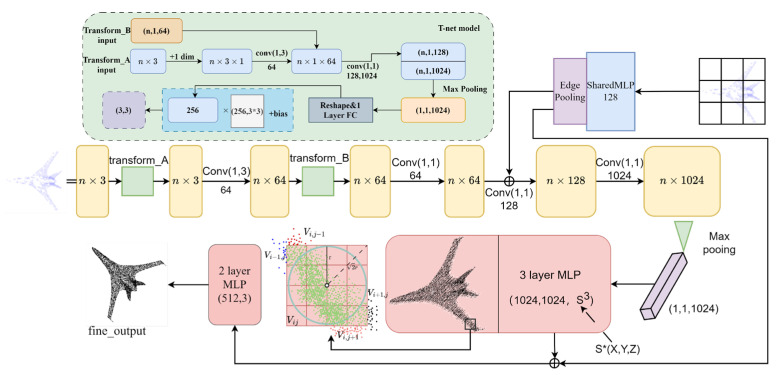
It shows the structure of our proposed point cloud completion model, which is inspired by the PointNet architecture. The model takes global and local feature representations as input and generates a set of points as output. Our approach combines global and local point cloud density with spatial distance for denoising optimization. The figure also illustrates the transformation networks A and B in our model, hereafter referred to as transform_A and transform_B. S* represents a set of coordinates at any position.

Overall, our proposed approach achieves state-of-the-art results on several benchmark datasets for point cloud completion tasks. Our method provides significant implications for applications in robotics, autonomous driving, and 3D modeling, improving the accuracy and efficiency of point cloud completion tasks. We believe that our approach can serve as a useful foundation for future research, contributing to the development of advanced algorithms for point cloud analysis and processing. Our overall structure is shown in Figure 1.

### 3.1. Regional Experience Field

**Figure 2:**
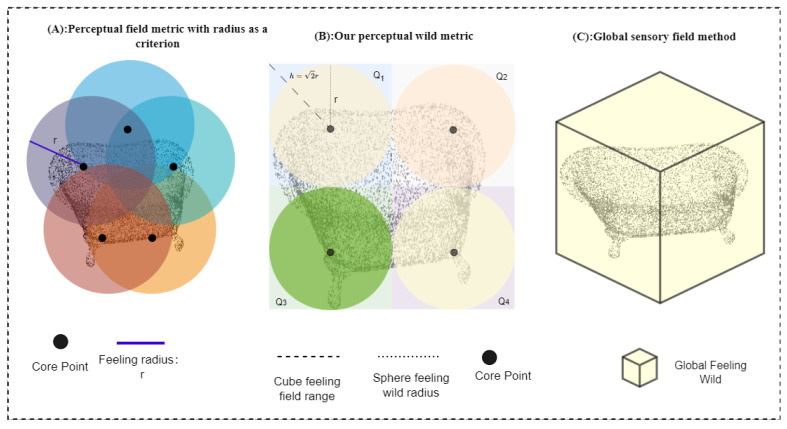
Comparison of collection methods in different sensory fields. (**A**). Perceptual field metric based on the radius criterion is the most commonly used method among researchers. It focuses on capturing local information within a specific radius. (**B**). Our proposed approach combines the perceptual field metric with the global sensory field approach. By incorporating two 3D geometric forms, we enhance the perception of the local field, resulting in a more comprehensive understanding of the data. (**C**). The global sensory field approach, although valuable for capturing global information, is used less frequently due to its high parametric characteristics, which present computational challenges.

To avoid the problems of distortion of the simplified point cloud structure and reduction of data volume, we do not cluster the delineated regions, allowing us to extract features more directly. We list the three dominant methods for sensory field delineation, including the structure proposed in this study. As shown in Figure 2, In Figure 2A, the perceptual field metrics are based on the radius criterion. This method primarily focuses on the local regions within a certain radius around each point, enabling the capture of local details and structural information. However, the performance of this method may be impacted by the challenge of finding a suitable universal radius that applies to all scenes, as the radius size needs to be specified in advance. Additionally, the emphasis on local regions may result in inadequate capturing of the overall point cloud structure and global features. In Figure 2B, we combine the perceptual field metric with the global sensory field approach and use two three-dimensional geometric forms to enhance the perception of the local field. Combining the radius-based point cloud receptive field and the global point cloud receptive field-based method can obtain a more comprehensive point cloud perception. By considering both local and global receptive fields, the local details and global structural information of the point cloud can be fully utilized to improve the richness of the perception. Combining the two receptive field methods can compensate for their respective shortcomings, e.g., the local receptive field can capture the local details while the global receptive field can consider the global structure, thus improving the robustness to noise and anomalies. At the same time, this method needs to adjust and optimize the corresponding parameters, including radius size, weights, etc., which may require more experiments and debugging. In Figure 2C, the global sensory field approach is used alone. In contrast, the use of a global point cloud perception field is not constrained by a specific radius. It allows for considering the global structure and features of the entire point cloud, leading to better capture of the overall information. However, this approach requires larger computational and storage resources to handle the global point cloud features, and it is also more sensitive to noise and outliers.

Given a 3D point cloud object with N points, where each point’s attributes describe its coordinates $\left(x_{n},y_{n},z_{n}\right)$ and RGB color information $\left(R_{n},G_{n},B_{n}\right)$, we define the set of tangent points $Q=\{Q_{m}|m=1,2,...M\}$ and $C=\{C_{m}|m=1,2,...M\}$. The set Q denotes the regular rectangular tangent region and C denotes the sphere region formed by the core point k and the radius r. Although point cloud data is not defined on a regular grid, the space occupied by its samples is fixed. We define the space occupied by a set of point clouds as S, where S={Sx,Sy,Sz}, and since all points are normalized to be between −1 and 1. So, $S\in \left[-1,1\right]$. We divide S uniformly and set the size as V. We define a point K as the kernel point in Q, and k is at the center of mass of the cubic tangent region Q. Q shares a k with C. The tangent point set Q is the same as the inner core of the point cloud cluster C. The points that fall inside this sphere will be the neighbor points of point k and participate in the feature calculation of k. The neighboring [31,32,33] points of k are defined as all the point cloud points distributed inside the cube and sphere with k as the core.

In this paper, where D is the relative distance between the point cloud core (x,y,z) and other scattered points $\left(x_{i},y_{i},z_{i}\right)$ in a uniformly tangent cube. However, in noisy, incomplete, and irregularly distributed initial point clouds x, it is difficult to perfectly complement small structural features, especially those “false details” that can have a strong negative impact on internal perceptual feature learning. Traditional point cloud simplification algorithms may remove noisy points and reduce the number of point clouds but lose some geometric features. Therefore, we use a multi-region training strategy [34] to improve the CNN network with a special convolution for each cut small region independently. We region and cluster the initial point cloud based on density, dividing the point cloud into multiple local regions or clusters; extract specific small structural features within each local region to capture local details and structural information; and train and optimize independently for each local region. The multi-region training strategy can be iterated and tuned several times to further optimize the complementary small structural features.

### 3.2. Global and Sub-Regional Convolution

In a two-dimensional convolutional neural network, the convolution operation can be regarded as computing the similarity between the two-dimensional kernel and the associated image. A larger output value indicates a higher visual similarity. In a 3D point cloud, the convolution operation computes the similarity between a 3D kernel and the associated 3D data. The output value indicates the visual similarity, and we adjust the convolution result by combining the inverse density value F. However, unlike 2D CNNs [35,36], convolution in 3D structures is not a simple task because there is no one-to-one relationship between points in 3D structures. Although we use spherical and cubic shape sensing fields, relational connections are not effectively made at the connections of each cut-off region. Therefore, we designed a special network for extracting global features based on the PointNet network model. While PointNet uses distance metrics to partition the point set into local regions with overlap and extracts local features from small to large ranges to extract global features of the whole point set, it can still be overwhelming when dealing with complex point cloud completion tasks. It treats each point as an independent input and ignores the adjacency relationships and local structures between points, which may lead to difficulties in capturing details and local relationships when dealing with point clouds with complex local shapes and structures. The computational complexity increases as the size of the point cloud increases, and for large-scale point cloud data, this may lead to higher time and computational resource requirements for training and inference. To address the limitations of existing methods and to improve the accuracy of point cloud generation, we propose the architecture shown in Figure 1. The architecture takes inspiration from the PointNet model and uses a combination of local and global features to generate a set of output points. This approach uses a two-step process. First, in (a), we utilize a baseline depth architecture that decodes the potential global feature representation into a set of points of a specific size. This step serves as the basis for generating the initial point cloud. To improve the quality and detail of the generated point cloud, we introduce additional input points in (b) that are sampled uniformly in the unit cube. By introducing these additional points, we enable the model to capture more local information and finer details in the point cloud. In addition, we include a denoising optimization step in (c) that combines the global-local point cloud density with the spatial distance. This helps to further refine the generated point cloud by reducing noise and improving the consistency of the overall structure. To facilitate the transformation and alignment of the point clouds, we introduce the structures transform_A and transform_B. These structures serve as miniaturized PointNet models that take the input point cloud as input and output a 3 × 3 transformation matrix. This transformation matrix is then applied to the input points to align them in a normalized coordinate system. This transformation/alignment network plays a key role in normalizing rotations, translations, and other variations within the point cloud, resulting in a more accurate and aligned point cloud output.

### 3.3. Sub-Regional Optimization

Global features and local features have different importance in expressing different aspects of the point cloud. If the weights of these features are not properly balanced during the fusion process, or if some specific global or local features are too prominent, it can easily lead to point cloud overflow phenomena. For example, too strong global feature weighting may lead to over-expansive point cloud regions, while too strong local feature weighting may lead to too dense local point clouds.

Although our method optimizes the local point cloud and detailed structure, it generates some discrete points and noise compared with the traditional point cloud processing methods. During the fusion process, if the perception of the local area is insufficient, i.e., the edge and detail information of the local point cloud cannot be captured accurately, it may lead to the point cloud overflow phenomenon. This may be because the perception of fine structures or edges in the point cloud is not sharp enough in the local feature extraction or fusion process, resulting in too many or abnormally dense points in the generated point cloud. To solve the point cloud overflow phenomenon, we need to pay attention to balancing the weights of different features in the process of global feature and local feature fusion and ensure the accuracy and non-redundancy of the features. Meanwhile, to improve the perceptiveness and accuracy of the local area, the point cloud overflow phenomenon can be improved by adjusting the size of the perceptual domain and increasing the resolution of the perceptual domain. In addition, appropriate point cloud sampling and noise processing methods can also help reduce the discrete points and noise from the point cloud overflow phenomenon.

Therefore, we design an optimization structure at the end of the network to learn how to combine features of different region sizes to obtain a robust result, to eliminate unnecessary point cloud overlap and point cloud overflow from local to local. We define the “inverse density” of the regional point cloud as $\rho_{i}$, and the total density of the point cloud as ρ. The inverse density can be defined as the ratio of the number of points in the neighborhood around P to the volume of the neighborhood. The volume of the neighborhood can be determined by the radius of the neighborhood. A higher inverse density indicates a denser regional distribution in the neighborhood of the region; conversely, a lower inverse density indicates a sparser regional point distribution in the neighborhood of the region. where:

(1) $$\rho_{i}=\frac{n-n_{i}}{S-S_{i}},\rho =\frac{n}{S}$$

where ρ denotes the point cloud density, n is the total number of point clouds, $n_{i}$ is the number of point clouds in the i-th tangent region, S is the point cloud size, and $S_{i}$ is the size of the i-th tangent region. By calculating the inverse density value of the current region, the region division strategy can be adjusted to ensure that the size of the divided region is suitable. When there is a significant difference between the inverse density value and the density value, it may be necessary to consider the problem of too large or too small region delineation and adjust the size of the delineated region accordingly. Through several iterations, the delineation strategy can be gradually adjusted to obtain appropriate region delineation results. High inverse-density values and low-density values indicate that the distribution of points in the region is relatively sparse, and the delineated region is too large, which may lead to excessive diffusion or loss of local details. On the contrary, low inverse-density values and high-density values indicate that the distribution of points in the region is relatively dense and the divided region is too small, which may lead to merging or omission of details. In this case, try to increase the size of the delineated region to better balance the global features and local details.

By iteratively adjusting the size of the delineated region and appropriately adjusting the relationship between the inverse density value and the density value, a suitable region delineation can be obtained to better solve the problem of overflow and irregular distribution in the point cloud. This can preserve the details and structural information of the point cloud and improve the effect of point cloud optimization.

**Figure 3:**
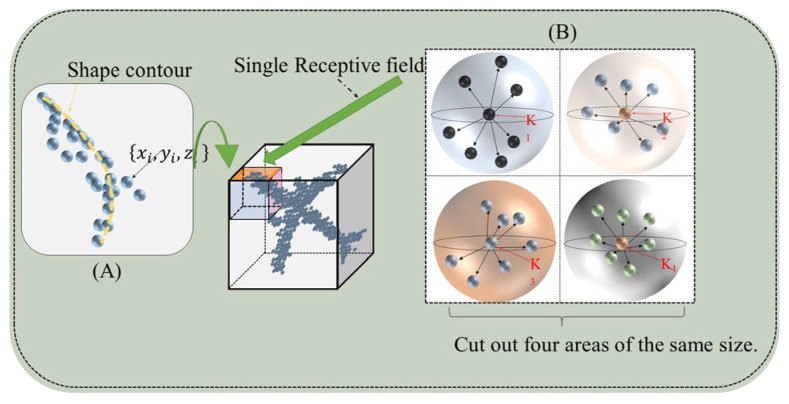
Diagram showing subregion optimization in point cloud refinement. The cube is divided into several subregions, each with cores representing its regional perceptual field. To achieve better refinement results, only the discrete points of each region are predicted in the refinement block. (**A**) shows the structural shape of point clouds in a single differentiated region, with yellow dashed lines explaining the simplified structural lines of point clouds in this region. (**B**) shows the segmentation method in the segmentation region, where the center point K is the center of the sphere. The number of segmentation regions can be determined based on the actual situation and experimental results. The figure shows the effect of dividing a small region into four regions.

To keep the details of the output results, we predict only the discrete points of each region in the refinement block. This method allows us to focus more on local details. By predicting only the discrete points, we can reconstruct the local structure more accurately and retain the detailed information in the original point cloud. This helps improve the ability of the model to complement the small structural features in the point cloud. The sub-regional optimization concept is shown in Figure 3, it illustrates the concept of subregion optimization used in our proposed point cloud refinement method. As shown in (B), the cube is divided into several subregions, each of which has several cores k representing its regional perceptual field core. To keep the output results detailed, as shown in (A), we predict only the discrete points of each region in the refinement block. This allows us to perform more targeted optimization and achieve better refinement results. Therefore, we consider a combination of the DBSCAN [37] (Density-Based Spatial Clustering of Applications with Noise) algorithm and the partition-based distance density function for point cloud optimization. Combining the DBSCAN algorithm with a partition-based distance density function and selecting density-based centroids as key points in the optimization process can effectively capture important structures and features in point clouds. This approach utilizes the density information of point clouds and reduces the influence of noise, thereby improving the effectiveness of point cloud optimization. By calculating the density around each point, we can determine the distribution of points in different regions of the point cloud. This enables us to identify regions with higher densities, which often represent important structures or features in the point cloud. Selecting density-based centroids as representatives of regions is a deliberate choice that better captures the characteristics of the entire region. Centroids, computed as the average position of all points in a region, are more likely to fall in the center or areas with higher density. This choice enables the centroids to better represent the features of the entire region while minimizing the impact of isolated or noisy points.

Furthermore, selecting density-based centroids helps effectively reduce noise in the output results. By excluding points with lower densities as centroids, we can filter out the potential noise or less significant points, thereby improving the quality and accuracy of the output results. In summary, combining the DBSCAN algorithm with a partition-based distance density function and selecting density-based centroids as key points in the optimization process effectively captures important structures and features in point clouds. This approach fully leverages the density information of point clouds and reduces the impact of noise, resulting in improved point cloud optimization. We select centroids by (the density-based centroid selection function), and this centroid can partially overlap with the k approximation when the perceptual field division is clear enough. Unlike the traditional clustering approach, we only do the removal process for point clouds that are far from the density center. At this stage, the k in the tangent region is no longer restricted to one but uniformly dispersed j, which is set to $k_{j}$, and the dispersion is uniformly dispersed at standard intervals in three-dimensional space, $j=v^{2}$. We calculate $\rho_{j}$ with $k_{j}$ as the core and $\frac{r}{v}$ as the radius. The advantage of using standard spacing is the ability to provide a consistent segmentation method while maintaining the overall point cloud structure. In this way, density and structural variations in different point cloud regions can be reasonably captured without some regions being over or under-covered due to uneven segmentation.

In addition, the choice of uniformly dispersed standard intervals can simplify the computation and processing. Due to the consistency of the standard spacing, we can more easily define and calculate the relevant properties, such as density, radius, etc., for each subregion. This makes the algorithm implementation and optimization process more efficient.

(2) $$\rho_{j}=\frac{n_{j}}{\frac{3\pi r^{2}}{v^{2}}}=\frac{n_{j}v^{2}}{3\pi r^{2}}$$

(3) $$RM_{pre}=max(|\rho_{j}-{\bar{\rho }}_{j}|)$$

(4) $$RM=H(LQ,LC)=\left\{Maxd(k_{j},x_{i})|x_{i}\in Remove_{pre}\right\}$$

(5) $$d(p,q)=\sqrt{\sum_{i=1}^{n}{\left(p_{i}-q_{i}\right)}^{2}}$$

The spatial representation in our density representation uses a spatially divided regional volume representation, as shown in Equations (2)–(4) represents the process of preprocessing the point cloud in the case of inhomogeneous density, where $RM_{pre}$ denotes the operation of removing discrete points from the region, and RM is the removal of individual discrete points from the overall point cloud after deprocessing the three points. Equation (5) allows us to go to individual discrete points for removal based on the formula, i.e., spatial distance.

## 4. Experiments and Evaluations

### 4.1. Loss Function

The training loss of the model consists of two parts [38]: local enhancement LA and fusion refinement LS. Among them, locally enhanced LA has two loss terms, core divergence loss $LA_{1}$ and local enhancement reconstruction loss $LA_{2}$, where $LA_{1}$ is:

(6) $$LA_{1}=D_{KL}(Q\|C)=\sum Q_{i}\times \log\frac{Q_{i}}{C_{i}}$$

$LA_{2}$ is a local enhancement loss function, and it is worth noting that when the focus is not on the overall point cloud, but on each point cloud kernel, the decision boundary of the object surface we focus on is no longer too complex to fit with neural network methods. The first is to predict the gap between the sample value and the true value, and the design loss function $L(\left\{S_{i}^{zq}\right\},\left\{S_{i}^{tr}\right\})$ is used to perform this task. It can be expressed as:

(7) $$L(S_{i}^{z}q,S_{i}^{t}r)=d(S_{i}^{z}q,S_{i}^{t}r)$$

where i is the number of point cloud kernels, and $S_{i}^{zq}$ and $S_{i}^{tr}$ represent the predicted and true values of the sample, respectively. We refer to the spatial distance formula to define the distance between $S_{i}$ as:

(8) $$\tau ={\sum_{y\in S_{i+1}}\min_{x\in S_{i}}\left\|x-y\right\|}_{2}^{2}$$

(9) $$d(S_{i},S_{\left(i+1\right)})={\sum_{x\in S_{i+1}}\min_{y\in S_{i}}\left\|x-y\right\|}_{2}^{2}+\tau$$

where x represents the set of predicted points and y represents the set of true points. x and y are the core structure of the point cloud after slicing the point cloud.

For each point in the true and predicted sets, function d finds the nearest neighbor in both sets and squares the distances. As a function of the midpoint position of $S_{i}$ and $S_{i+1}$, this function is continuous and piecewise smooth. The range search for each point is independent and therefore parallelizable.

Since the relative position of x=(0,0,0) is set separately in each point cloud core and the core point during the prediction process, the relative displacement of this position may occur when comparing the same point cloud core of different point clouds, so the prediction loss function of the center point x of the point cloud kernel becomes extremely important, we design:

(10) $$F_{\theta }(p_{ij},x_{i})=d(p_{ij},x_{i})$$

(11) $$L_{G}(\theta )=\frac{1}{\left|G\right|}\sum_{i=1}^{\left|G\right|}\sum_{i=1}^{k}H(F_{\theta }(p_{ij},x_{i}),t_{ij})$$

where $x_{i}$ is the absolute position value of the ith point cloud core of batch *G*, $t_{ij}$ is the core position of the real point cloud, $p_{ij}$ is the distance value from the core point of the point cloud to the core collection, and H is the cross-entropy loss. Therefore, we specify the local enhancement loss function. where $LA_{2}$ is:

(12) $$LA_{2}=L\left(\left\{S_{i}^{zq}\right\},\left\{S_{i}^{tr}\right\}\right)+W\cdot L_{G}(\theta )$$

where w is the parameter that can be learned by the neural network, which is used to linearly adjust the parameter change of the distance from the core point of the point cloud to the core set. 

In this study, we use distance to solve the assignment problem, resulting in the optimal bias φ being a unique constant for all point sets except for the zero measurement subset. Although deep graph convolutional networks have good expressiveness, they still face uncertainty in predicting the detailed geometry of 3D objects, which may arise from limited network capacity, input resolution size, or information loss in the residual cloud. To address this issue, we adopt the cross-entropy loss function(LS) to optimize the distance between classes.

(13) $$L_{S}=-\sum_{i=1}^{m}\log\frac{\chi }{\sum_{j=1}^{n}\beta },\beta =e^{\left(w_{\left(y_{i}\right)}^{T}+b_{y}i\right)},\chi =e^{\left(w_{\left(y_{i}\right)}^{T}x_{i}+b_{y}i\right)}$$

The $x_{i}\in R^{d}$ represents the i-th deep feature, which belongs to the i-th sharded point cloud core. d is the feature dimension. $w_{j}\in R^{d}$ represents the j-th column of the weights $w\in R^{d\times n}$ in the last fully connected layer, and $b\in R^{n}$ is the biased term. The size of the mini-batch is m, and the number of classes is n. The joint loss function can be expressed as $L=LA_{1}+LA_{2}+L_{S}$.

### 4.2. Implementation Details

In this section, we present the qualitative and quantitative results of our method for 3D point cloud completion and compare them with several benchmark models on the dataset: ShapeNet.

ShapeNet [39] is a large online repository of 3D shapes created by Stanford University and Princeton University. It contains various 3D objects from different categories such as furniture, cars, airplanes, and animals. The data also includes annotations for each object, such as category labels, split masks, and surface normal. ShapeNet can be used for a variety of tasks such as 3D object recognition, 3D shape search and retrieval, 3D model composition, and 3D printing. We will show the comparison results of some mainstream point cloud completion task models under the same dataset. Because of the disorderly nature of point clouds, we must find a suitable way to measure the quality difference between generated point clouds and ground truth point clouds. There are two measures CD (chamfer distance), and EMD (earth mover’s distance) [40].

(14) $$\phi =\frac{1}{\left|Q_{1}\right|}\sum_{x\in Q_{1},y\in Q_{2}}\min{\left\|x-y\right\|}_{}^{2}+\frac{1}{\left|Q_{2}\right|}\sum_{x\in Q_{1},y\in Q_{2}}\min{\left\|y-x\right\|}_{}^{2}$$

(15) $$C\hat{D}=\frac{1}{\left|S_{1}\right|}{\sum_{\phi_{1}\in S_{1},\phi_{2}\in S_{2}}^{\min}\left\|\phi_{1}-\phi_{2}\right\|}^{2}+\frac{1}{\left|S_{2}\right|}{\sum_{\phi_{2}\in S_{2},\phi_{1}\in S_{1}}^{\min}\left\|\phi_{2}-\phi_{1}\right\|}^{2}$$

We train all models for 300 epochs, with a batch size of 16, a learning rate of 0.01 or 0.015 depending on stability, and an Adagrad optimizer [41]. We evaluate our model on some classes of the ShapeNet dataset and compare it with state-of-the-art point cloud completion methods. For each class, we calculate the CD (averaging over the number of class instances) and EMD [42] distance. The evaluation results of our method and the most advanced method [43] are shown in Table 1 and Table 2. The excellent results in the table will be displayed in bold text.

We performed a random sparse operation on the number of point clouds under the uniform dataset to obtain 80%, 50%, and 30% of the number of point clouds in the original dataset. It is worth noting that the average results we obtained after three random sparse operations are shown in Table 3. At the time when the data reached 30%, several comparison models and text accounted for making the models no longer have normal prediction results. This is probably because the point cloud in this state no longer has any expressible feature shape and content. 

### 4.3. Optimization

Point clouds are widely used in various fields such as robotics, autonomous driving, and 3D modeling. However, due to the discrete nature of the data, point clouds are often accompanied by noise and redundant points, which can interfere with accurate processing and analysis. To address this issue, we propose the use of point cloud density and clustering algorithms to effectively remove noise and refine the point cloud structure. Specifically, we use inverse density values to weigh each point and employ density-based clustering algorithms to group nearby points into clusters. This approach not only reduces noise but also preserves important structures and shapes of the original point cloud. We evaluate our method on benchmark datasets and demonstrate its effectiveness in improving the accuracy and efficiency of point cloud completion tasks. 

Our goal is to find a function f that maps a point cloud containing noise and outliers to a clean and uniformly distributed point cloud. To achieve this goal, we can use the following steps:

(1) First, we need to estimate the local density of each point $P_{i}$, i.e., the number of points in its r-neighborhood. We can use either the k-nearest neighbor algorithm or the mesh partitioning algorithm to achieve this step.
(2) Then, we can determine which points are outliers or noise points based on the density threshold and remove them. This step can be represented by the following equation: $\tilde{o_{i}}=g(P_{i})$. where $\tilde{o_{i}}$ is the probability that $P_{i}$ belongs to an outlier, g is a classification function, and $P_{i}$ is the set of points in the r-neighborhood of $P_{i}$. We define V uniformly dispersed core points $\hat{k}$ in the tangent region, with the distance between $\hat{k}$ and $\hat{k}$ defined as $\hat{r}$, we need to estimate the local density of each core point $\hat{k}$ and find the n farthest distance points with the distance limit (n is determined empirically).
(3) The m farthest points from the cores of the cat-off region are defined as “potentially discrete points”. The region represented by each $\hat{k}$ is sampled or filtered to reduce the amount of data and preserve the main features. We can use uniform sampling, nearest neighbor interpolation, bilateral filtering, etc. to achieve this step. This step can be represented by the following equation: $d_{i}=f({\hat{P}}_{i})$, where $d_{i}$ is the offset estimated for the point $P_{i}$ (or zero if there is no offset), and f is a denoising or refinement function that ${\hat{P}}_{i}$ is the other points in the cluster to which $P_{i}$ belongs.
(4) The final denoised and refined point cloud is obtained as: ${\tilde{P}}_{i}={\hat{P}}_{i}-d_{i}$, where ${\tilde{P}}_{i}$ is the final prediction for point $P_{i}$. The comparison results of the point cloud detail optimization after our design are shown in Table 4. The CD-D(CD With Density) and EMD-D(EMD With Density) are representations of the results after the optimization of our model combining density and spatial location.

### 4.4. Results

**Figure 4:**
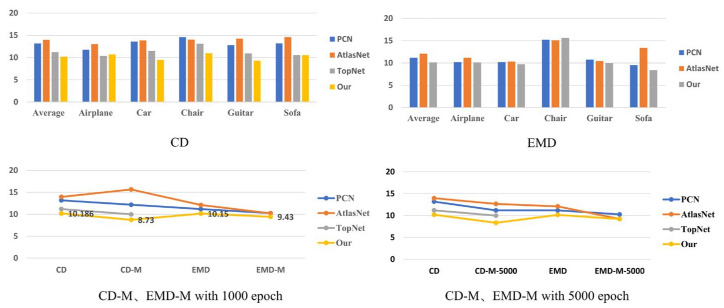
Test results score schematic chart. In this figure, we analyze the precision test results of different models and our proposed model in terms of CD and EMD metrics. We also compare the results of integrating our point cloud density refinement module (CD-D, EMD-M). Furthermore, we compare the accuracy of 300 and 3000 iterations.

The final denoised and refined point cloud is obtained as: ${\tilde{P}}_{i}={\hat{P}}_{i}-d_{i}$, where ${\tilde{P}}_{i}$ is the final prediction for point $P_{i}$. The overall results are shown in Figure 4. After applying the point cloud density refinement module for 300 iterations, our model showed a significant improvement in CD accuracy, with a decrease of 1.456. Furthermore, in 3000 iterations, the CD value decreased by 0.39. Similarly, we observed a decrease in EMD accuracy from 10.15 to 9.43 in 300 iterations, and to 9.2 in 3000 iterations. These results demonstrate the effectiveness of our approach in refining and improving the accuracy of point cloud predictions. Our study demonstrates that the integration of the point cloud density refinement module not only enhances the performance of our proposed model, but also improves the accuracy of other models such as PCN, AtlasNet, and TopNet, as evidenced by the results presented in Table 1, Table 2 and Table 4. Furthermore, our findings suggest that increasing the number of iterations can significantly improve the accuracy of the point cloud density refinement module. These results have important implications for the development of more accurate and robust point cloud processing techniques. In Figure 4, we present a schematic chart that shows the precision test results of different models, including our proposed model, in terms of CD and EMD metrics. To further improve the accuracy of our proposed model, we also integrated a point cloud density refinement module (CD-D, EMD-M) and compare its results to those without the module. Additionally, we compare the accuracy of our proposed model after 300 and 3000 iterations. The results show that our proposed model with the density refinement module and 3000 iterations achieves the highest accuracy compared to the other models, as evidenced by the lower CD and EMD values. These findings indicate that our proposed model can effectively improve the precision of point cloud completion and optimization.

**Figure 5:**
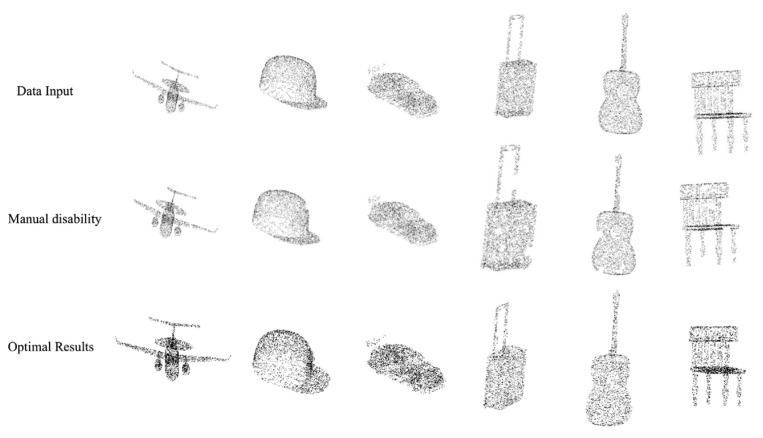
After multiple experimental comparisons, the experimental results of this model are good, with good completion ability for manually incomplete point clouds and strong optimization ability for overall point cloud density.

Through multiple experimental comparisons, the proposed model demonstrates a strong ability to complete manually incomplete point clouds and optimize overall point cloud density. The experimental results show that the model achieves good performance in terms of point cloud reconstruction and complete accuracy. Additionally, as illustrated in Figure 5, the proposed model effectively fills in the missing parts of the point cloud and generates more complete and accurate point cloud data. These findings suggest that the proposed model can be a promising solution for point cloud completion and optimization tasks.

## 5. Conclusions and Discussions

We propose a novel method for point cloud completion tasks that integrates density and partition-based techniques to generate accurate and efficient point cloud completion results. Our approach utilizes both global and local features, extracted from the original and locally divided point clouds, to capture the overall shape and fine-grained details of the object. The fusion of these features is performed by a convolutional neural network, resulting in robust and accurate completion results. To further refine the output, we utilize density-based clustering algorithms and inverse density values to effectively remove noise and improve the structure of the output point cloud. Invariance to rotation and translation is achieved by including a transformed network in our architecture. Our proposed approach achieves state-of-the-art results on several benchmark datasets for point cloud completion tasks, indicating its effectiveness in improving the accuracy and efficiency of point cloud processing in various fields such as robotics, autonomous driving, and 3D modeling. Our proposed approach presents several innovative contributions:

(1) Local-Global Fusion: We introduce the concept of perceptual fields, which divide the input point cloud into uniform local regions. By combining global and local information, our method effectively captures the overall shape and fine-grained details, preserving sharp edges and detailed structures.
(2) Transformer-based Model: We utilize a transformer model to process the feature vectors obtained from each perceptual field. This allows us to capture long-range dependencies and effectively infer missing elements in the point cloud.
(3) Geometric Density-aware Block: We design a geometric density-aware block to leverage the inherent 3D geometric structure of the point cloud. This block enhances the preservation of important geometric features and improves the accuracy of the completed point cloud.

However, there are also some limitations to our proposed approach:

(1) Lack of scalability: Our proposed method may face scalability issues when dealing with large-scale point clouds, as it involves the partitioning of the point cloud.
(2) Limited effectiveness for complex objects: While our method achieves state-of-the-art results on several benchmark datasets, it may have limited effectiveness for complex objects with more intricate shapes and details.
(3) Limited generalizability: Our approach may have limited generalizability to point clouds from different domains or with different characteristics, as it was designed specifically for point cloud completion tasks.

In conclusion, our proposed method represents a significant step towards more accurate and efficient point cloud processing. While there are limitations to our approach, we believe that our contributions can have a positive impact on various applications that rely on 3D data processing. Further research is needed to address the limitations of our proposed approach and explore its potential for broader applications.

## Figures and Tables

**Table 1:** Point cloud completion results with various models. the CD loss is multiplied by 10^4^. (lower is better).

Method	Avg.	Airplane	Car	Chair	Guitar	Sofa
PCN	13.17	11.74	13.56	14.58	12.79	13.2
AtlasNet	13.96	13.01	13.85	14.03	14.26	14.56
TopNet	11.184	**10.4**	11.5	13.08	10.93	10.55
**Our**	**10.186**	10.7	**9.45**	**10.95**	**9.3**	**10.53**

**Table 2:** Point cloud completion results with various models. the EMD loss is multiplied by 10^3^. (lower is better).

Method	Avg.	Airplane	Car	Chair	Guitar	Sofa
PCN	11.18	10.2	10.2	15.22	10.76	9.53
AtlasNet	12.097	11.16	10.3	**15.1**	10.43	13.4
**Our**	**10.15**	**10.15**	**9.73**	15.6	**9.98**	**8.41**

**Table 3:** Point cloud completion results with various models. the CD loss is multiplied by 10^4^. (lower is better). The number of points clouds is abbreviated as NOP (number of points). NOP*n% means n percent of NOP.

Method	NOP*80%	NOP*50%	NOP*30%
PCN	14.86	**27.95**	null
AtlasNet	15.09	29.56	null
TopNet	13.012	37.69	null
**Our**	**12.007**	28.82	null

**Table 4:** Comparison of point cloud completion under different models and optimized point cloud completion.

Method	CD	CD-D	CD-D 3000	EMD	EMD-D	EMD-D 3000
PCN	13.174	12.174	11.174	11.18	10.25	10.25
AtlasNet	13.96	15.66	12.66	12.097	10.21	9.21
TopNet	11.184	9.98	9.98	NULL	NULL	NULL
Our	**10.186**	**8.73**	**8.34**	**10.15**	**9.43**	**9.2**

## Data Availability

We use the public dataset: Shapenet, which can be found on https://shapenet.org/ (accessed on 1 March 2023).
